# 

**Published:** 1898-02

**Authors:** 


### CONTENTS.

ORIGINAL COMMUNICATIONS—

Quinine in Malaria, Excluding the Simple Intermittents. By J. G. Van Marter, Jr., M.D., Savannah, Ga. .......... 793

The Effect of Intense Flashes of Electric Light Upon the Eye. By Dunbar Roy, A.B., M.D., Atlanta, Ga. .......... 802

The Treatment of Gonorrhœa by Hydrostatic Irrigation. By W. L. Champion, M.D., Atlanta, Ga. .......... 810

Puerperal Infection. By St. Joseph B. Graham, M.D., Savannah, Ga. .......... 813

A New Form of Treatment in Uterine Disease. By Herman D. Marcus, M.D., Philadelphia, Pa. .......... 818

SOCIETY REPORTS—

Clinical Society of St. James Dispensary .......... 821

CORRESPONDENCE—

Criminal Abortion—Information Wanted .......... 829

EDITORIAL—

Medical Matters in 1897 .......... 831

Successful Esophago-Enterostomy .......... 834

The Board of Health and Dr. Olmsted's Bill .......... 836

MEDICAL ITEMS .......... 838

BOOK REVIEWS .......... 846

SELECTIONS AND ABSTRACTS .......... 852

MEDICAL ITEMS—Continued .......... x, xii, xiii, xiv, xvi, xvii

ADVERTISERS' INDEX TO ADVERTISEMENTS .......... xviii

CLASSIFIED INDEX TO ADVERTISEMENTS .......... xxxiv

